# Gene expression profiles of human endometrial cancer samples using a cDNA-expression array technique: assessment of an analysis method

**DOI:** 10.1054/bjoc.2000.1238

**Published:** 2000-06-15

**Authors:** E Smid-Koopman, L J Blok, S Chadha-Ajwani, T J M Helmerhorst, A O Brinkmann, F J Huikeshoven

**Affiliations:** Department of Gynaecology and Obstetrics, University Hospital Rotterdam, PO Box 2040, Rotterdam, CA, 3000; Department of Pathology, University Hospital Rotterdam, PO Box 1738, Rotterdam, DR, 3000; Department of Endocrinology and Reproduction, Erasmus University Rotterdam, PO Box 1738, Rotterdam, DR, 3000, The Netherlands

**Keywords:** cDNA expression array, genes, endometrial cancer

## Abstract

The recently developed cDNA expression array technique can be used to generate gene-expression fingerprints of tumour specimens. To gain insight into molecular mechanisms involved in the development and progression of cancer, this cDNA expression array technique could be a useful tool, however, no established methods for interpreting the results are yet available. We used the Atlas cancer cDNA expression array (Clontech, USA) for analysing total RNA isolated from four human endometrial carcinoma samples (two cell-lines and two tissue samples), one benign endometrial tissue sample and a human breast cancer cell-line, in order to develop a method for analysing the array data. The obtained gene-expression profiles were highly reproducible. XY-scatterplots and regression analysis of the logarithmic transformed data provided a practical method to analyse the data without the need of preceding normalization. Three genes (Decorin, TIMP3 and Cyclin D1) were identified to be differentially expressed between the benign endometrial tissue sample and the endometrial carcinoma samples (tissue and cell-lines). These three genes may potentially be involved in cancer progression. A higher degree of similarity in gene-expression profile was found between the endometrial samples (tissue and cell-lines) than between the endometrial samples and the breast cancer cell-line, which is indicative for an endometrial tissue-specific gene-expression profile. © 2000 Cancer Research Campaign


*British Journal of Cancer* (2000) **83**(2), 246–251
© 2000 Cancer Research Campaign
doi: 10.1054/ bjoc.2000.1238, available online at http://www.idealibrary.com on IDEAL®

### Gene expression profiles of human endometrial cancer samples using a cDNA-expression array technique: assessment of an analysis method

**E Smid-Koopman[1], LJ Blok[3], S Chadha-Ajwani[2], TJM Helmerhorst[1], AO Brinkmann[3] and FJ Huikeshoven[1]**

[1]Department of Gynaecology and Obstetrics, University Hospital Rotterdam, PO Box 2040, 3000 CA Rotterdam; [2]Department of Pathology, University Hospital Rotterdam, PO Box 1738, 3000 DR Rotterdam; [3]Department of Endocrinology and Reproduction, Erasmus University Rotterdam, PO Box 1738, 3000 DR Rotterdam, The Netherlands

**Summary** The recently developed cDNA expression array technique can be used to generate gene-expression fingerprints of tumour specimens. To gain insight into molecular mechanisms involved in the development and progression of cancer, this cDNA expression array technique could be a useful tool, however, no established methods for interpreting the results are yet available. We used the Atlas cancer cDNA expression array (Clontech, USA) for analysing total RNA isolated from four human endometrial carcinoma samples (two cell-lines and two tissue samples), one benign endometrial tissue sample and a human breast cancer cell-line, in order to develop a method for analysing the array data. The obtained gene-expression profiles were highly reproducible. XY-scatterplots and regression analysis of the logarithmic transformed data provided a practical method to analyse the data without the need of preceding normalization. Three genes (Decorin, TIMP3 and Cyclin D1) were identified to be differentially expressed between the benign endometrial tissue sample and the endometrial carcinoma samples (tissue and cell-lines). These three genes may potentially be involved in cancer progression. A higher degree of similarity in gene-expression profile was found between the endometrial samples (tissue and cell-lines) than between the endometrial samples and the breast cancer cell-line, which is indicative for an endometrial tissue-specific gene-expression profile. © 2000 Cancer Research Campaign

**Keywords:** cDNA expression array; genes; endometrial cancer

Tumour development and progression involves a cascade of genetic alterations (Fearon and Vogelstein, 1990; Knudson, 1993). Techniques frequently used to study gene expression alterations, such as RT-PCR, differential display PCR and Northern blot analysis, have their limitations: some need large amounts of RNA, others are time-consuming and can only study a small number of genes simultaneously. With the development of the cDNA expression array technique, a method has become available to study the expression levels of a large range of genes in one single hybridization, requiring only a small amount of total RNA. Using this technique, a gene-expression fingerprint of every single tissue sample can be made. Being a novel technique, no established methods for interpreting the results are yet available. In recent publications mostly two single hybridization experiments are compared and differentially expressed genes are identified. Little attention is paid to the method which is used to analyse the data (Shim et al, 1998; Kaiser et al, 1999; Hoch et al, 1999). However, two research groups have described methods to analyse the array data: Hilsenbeck et al (1999) introduce a statistical method to compare more than two hybridization experiments and to identify differentially expressed genes, using a principal components analysis of mean-centred log-transformed data. Rhee et al (1999) analyse similarity in gene-expression patterns between three hybridization experiments using scatterplots and ranktests.

*Received 18 November 1999*
*Revised 2 March 2000*
*Accepted 10 March 2000*

*Correspondence to:* E Smid-Koopman

Endometrial cancer is the most common gynaecologic malignancy (Schottenfeld et al, 1995; Rose, 1996). In the majority of cases, approximately 75%, the tumour is confined to the uterus at time of diagnosis and has a relatively good prognosis. Patients with advanced/recurrent disease have a poor prognosis, with response rates to therapy of only 10–30% (Lentz, 1994; Rose, 1996). In general, tumour development and tumour progression are thought to be driven by genetic alterations. Therefore, insight into the molecular mechanisms involved in progression of endometrial carcinoma is important when searching for new tools to improve the outcome of patients with advanced/recurrent endometrial carcinomas. Using the cDNA expressing array technique, gene-expression fingerprints of a variety of endometrial carcinoma tissue samples can be produced, and differentially expressed genes can be identified. We used the Atlas Human Cancer Expression Array (Clontech, USA) on endometrial carcinoma tissue and cell-line samples. The analysis method and the results are evaluated in this article.

### MATERIALS AND METHODS

### Cell-lines

Ishikawa cells are derived from a human well differentiated endometrial adenocarcinoma and were a generous gift from Dr Masato Nishida (Tsukuba, Japan). ECC-1 cells are derived from a well differentiated adenocarcinoma of human endometrium transplanted to nude mice and were a generous gift from Dr PG Satyaswaroop (Hershey, USA). The T47D cells are derived from well differentiated human breast cancer and were a generous gift from Dr B van der Burg (Utrecht, The Netherlands). All cells were maintained in DMEM/F12 (Dulbecco's modified Eagle's medium/Ham's F12 (1:1 mix) with 15 mM Hepes, with L-glutamine) supplemented with penicillin/streptomycin and in the presence of 7.5% fetal calf serum and $10^{-9}$ M oestradiol in a 37°C incubator. For the experiments, cells were cultured to a 50% confluency, medium was changed to DMEM/F12 supplemented with 7.5% dextran-coated charcoal-treated fetal calf serum and $10^{-9}$ M oestradiol. Subsequently, cells were cultured for an other 72 h before harvesting. Total RNA was isolated as described below.

### Tissue samples

The endometrial carcinoma tissue samples were obtained from patients attending hospital for treatment of endometrial cancer (Department of Obstetrics and Gynaecology of the Academic Hospital Rotterdam and Department of Obstetrics and Gynaecology of the Sint Franscicus Hospital Rotterdam). The histological typing and grade were established by the Department of Pathology of the Academic Hospital Rotterdam according to the modified FIGO staging system (Mikuta, 1993). The two human endometrial carcinoma tissue samples used were tissue-sample 54: moderate well-differentiated (grade 2) endometrioid adenocarcinoma and tissue-sample 55: well-differentiated (grade 1) endometrioid adenocarcinoma. The benign human endometrial epithelial tissue sample was obtained from a patient attending the Department of Obstetrics and Gynaecology of the Academic Hospital Rotterdam for treatment of an uterus myomatosus (tissue-sample 36). The endometrial epithelial samples were excised directly after removal of the uterus from the body and frozen in liquid nitrogen. Sandwich sections were made, by the Department of Pathology of the Academic Hospital Rotterdam, to establish percentage of tumour in the studied samples. The carcinoma samples used in this study (samples 54 and 55) contained > 90% endometrial carcinoma tissue, the benign endometrial tissue sample (sample 36) contained > 90% benign endometrial epithelial tissue.

### RNA isolation

Total RNA was isolated from the cell-lines and the tissue specimen following the instructions of the protocol PT3231-1, advised to use in combination with the Atlas Human Cancer cDNA expression array by Clontech Laboratories Inc. (Palo Alto, California, USA).

### Atlas human cancer cDNA expression array

The Atlas human cancer cDNA expression array (7742–1) was purchased from Clontech Laboratories Inc. Two identical nucleic acid arrays were supplied containing 588 cancer-related human cDNAs spotted as duplicates on nylon membranes. A list of the genes spotted on the array, including array coordinates, is available at Clontech's web site (http://www.clontech.com).

### cDNA synthesis and hybridization

Total RNA was reverse-transcribed into cDNA using a mixture of array gene-specific primers and was labelled with $^{32}$P-dATP. Probe purification and hybridization to the array were performed following the array's user manual. The array was exposed to a phosphorimaging screen at room temperature for 24 h and scanned using the PhosphorImager (Molecular Dynamics, Sunnyvale, USA). The results were quantified using ImageQuant software (Molecular Dynamics). A grid was applied to the image of the blot to quantify the intensity of the hybridization of every spot. Background signal was defined as the average of the hybridization signals produced by the nine negative controls on the array. A gene was defined to be expressed when the hybridization signal extended to two times the background signal. According to the Array manufacturer (Clontech Laboratories), the radioactive cDNA signal is linear for RNAs present at levels of 0.01–3% of the total RNA population. Saturation of the hybridization signal was not observed.

### Statistic analysis

The expression data obtained from PhosphorImager analysis were logarithmic ($^{10}$log) transformed. The xy-scatterplot displays the expression levels of the genes for two compared experiments. Every dot represents one of the genes on the cDNA array. The x-value represents the $^{10}$log of the expression level of the gene in one experiment, the y-value represents the $^{10}$log of the expression level of the same gene in the other experiment. The xy-scatterplots were produced using Microsoft Excel 97 software.

The least-squares regression line $y = ax + b$ is the line with the smallest sum of squared vertical distances between the points of the xy-scatterplot and the line. The least-squared regression lines were drawn using Microsoft Excel 97 software.

R-squared value is the Pearson correlation coefficient of the least-squares regression line. The R-squared values were calculated using Microsoft Excel 97 software.

The standard deviation of the distance between the points and the least-squared regression line was calculated using Microsoft Excel 97 software. An area of standard deviation multiplied with 1.96 at both sides of the least-squared regression line was drawn in the xy-scatter to establish a 95% prediction interval. Genes located outside this area were identified as differentially expressed.

### RESULTS

Using a cDNA expression array technique we established the expression profile of 588 genes selected from different areas of cancer research in two human endometrial carcinoma cell-lines (Figure 1a and 1b), in two human endometrial carcinoma tissue samples (Figure 1d), in one benign human endometrial tissue sample (Figure 1c) and in one breast cancer cell-line. Using logarithmic-transformed raw data, obtained from phosphorimager analysis of cDNA expression array hybridization experiments, the gene-expression fingerprints of the different tissue and cell-line sample were compared. Using an xy-scatter plot, similarity in gene-expression fingerprints between the cell-lines and tissue samples was established and differentially expressed genes were identified.

### Normalization of the data

It seems that comparing gene-expressing fingerprints between cell-lines or tissue samples should be conducted only after normalization of the data for differences in background signal and for differences in intensity of hybridization. The cancer cDNA expression array contains nine genes known as housekeeping genes (liver

© 2000 Cancer Research Campaign

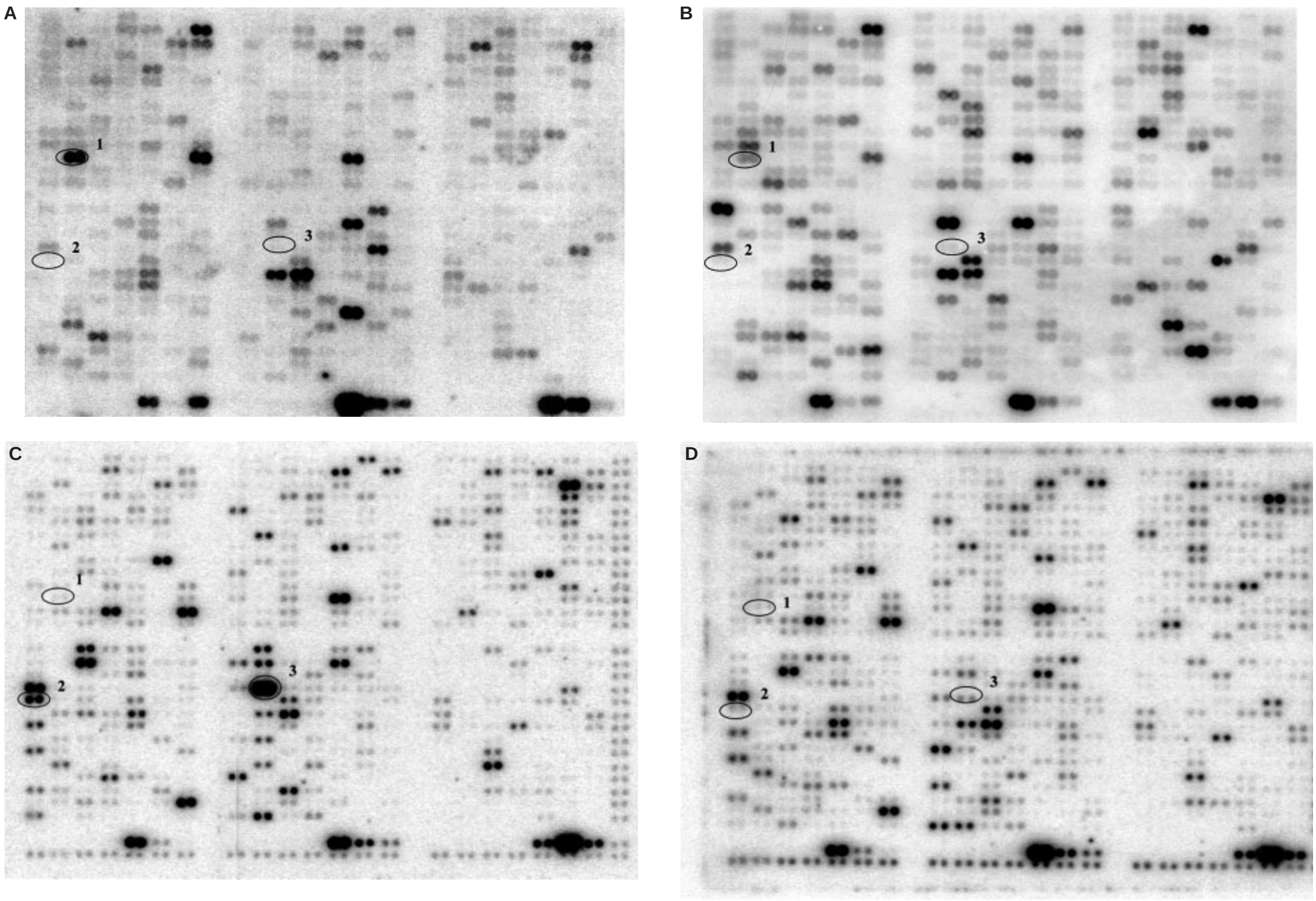

**Figure 1** Gene-expression fingerprints of endometrial cells. Atlas human cancer cDNA expression array (Clontech, USA) was hybridized with $^{32}$P-labelled cDNA probes obtained from total RNA of the Ishikawa endometrial carcinoma cell-line (A), the ECC-1 endometrial carcinoma cell-line (B), a benign human endometrial tissue sample (benign tissue sample 36) (C) and a well differentiated human endometrial carcinoma tissue sample (tumour sample 54) (D). The array contains 588 cancer-related human cDNAs spotted as duplicates. Nine housekeeping genes are spotted at the bottom line to serve as positive controls. Dark grey spots at the outer end of the array represent genomic DNA spots, which serve as orientation marks. 1 = Cyclin D1, 2 = Decorin, 3 = TIMP3.

**Table 1** R-squared values. Logarithmic transformed raw data were plotted in xy-scatter charts, regression analysis was performed and R-squared values were calculated using Microsoft Excel 97 software

| R-squared value | Ishikawa 1 | Ishikawa 2 | ECC-1 | T47D | Benign sample 36 | Tumour sample 54 | Tumour sample 55 |
|---|---|---|---|---|---|---|---|
| Ishikawa 1 | | 0.92 | 0.29 | 0.02 | 0.33 | 0.56 | 0.27 |
| Ishikawa 2 | 0.92 | | nd | nd | nd | nd | nd |
| ECC-1 | 0.29 | nd | | 0.33 | 0.22 | 0.26 | 0.42 |
| T47D | 0.02 | nd | 0.33 | | 0.06 | 0.05 | 0.16 |
| Benign sample 36 | 0.33 | nd | 0.22 | 0.06 | | 0.74 | 0.41 |
| Tumour sample 54 | 0.56 | nd | 0.26 | 0.05 | 0.74 | | 0.47 |
| Tumour sample 55 | 0.27 | nd | 0.42 | 0.16 | 0.41 | 0.47 | |

n.d. = not determined glyceraldehyde 3-phosphate dehydrogenase, tubulin alpha, HLA class 1 histocompatibility antigen C-4 α-chain, β-actin, 23-kDA highly basic protein, ribosomal protein S9, ubiquitin, phospholipase A2, HPRT). All these nine housekeeping genes, or a selection of these genes, could be used as standards in normalization. When using an xy-scatterplot and regression analysis, the raw data may be analysed without the need for preceding normalization of the data. In the least-squares regression line $y = ax + b$ the slope value a can be considered to represent the differences in hybridization intensity and the intercept value $b$ to represent the differences in background signal. Expression level of the highest and lowest expressed genes on the array varied extensively. Subsequently, when comparing expression data, the differences between genes with high expression levels will have much greater impact than the differences between low-expressed genes. Using a logarithmic transformation of the raw data equalized these differences to some extent.

*© 2000 Cancer Research Campaign*

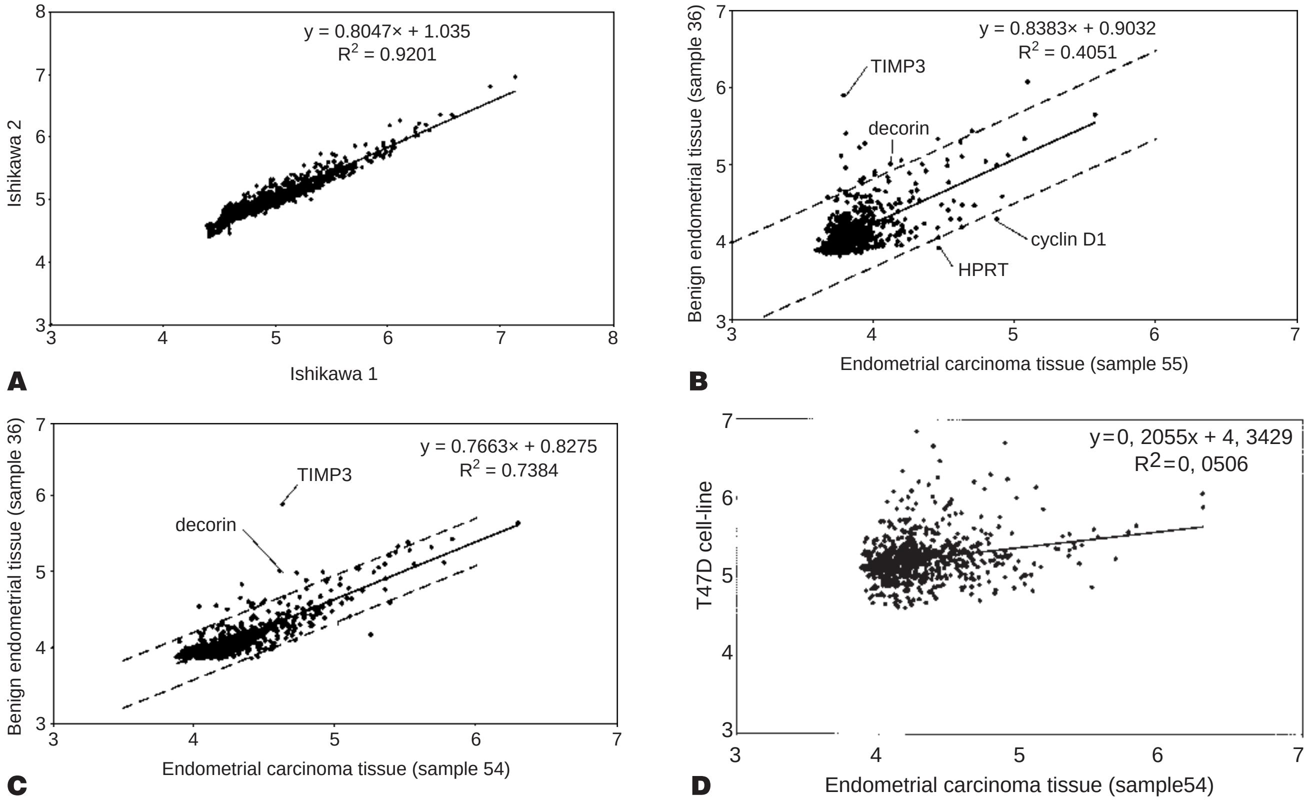

**Figure 2** Pair-wise comparison of gene-expression fingerprints using xy-scatterplot. A pair-wise comparison of the gene-expression fingerprint was made between: the two Ishikawa cell hybridizations to the twin array membranes (A), the benign human endometrial tissue sample 36 and the human endometrial carcinoma tissue sample 55 (B), the benign human endometrial tissue sample 36 and the human endometrial carcinoma tissue sample 54 (C), and between the T47D breast cancer cells and the human endometrial carcinoma tissue sample 54 (D).

**Table 2** Endometrial housekeeping genes and differentially expressed genes. Gene-expression was defined as a hybridization signal extending the value of two times the background signal and is represented by ×.

| | Ishikawa | ECC-1 | Tumour sample 55 | Tumour sample 54 | Benign sample 36 | T47D |
|---|---|---|---|---|---|---|
| Cyclin | × | × | × | × | × | – |
| CD 9 | × | × | × | × | × | – |
| GRB-2 isoform | × | × | × | × | × | – |
| c-myc binding protein | × | × | × | × | × | – |
| c-myc transcription factor (PUF) | × | × | × | × | × | – |
| BIGH-3 | × | × | × | × | × | – |
| Notch 2 | × | × | × | × | × | – |
| Hepatoma-derived growth factor | × | × | × | × | × | – |
| FAU | × | × | × | × | × | – |
| Cyclin D1; BCL-1 oncogene | × | × | × | – | – | × |
| Decorin | – | – | – | – | × | – |
| TIMP3 | – | – | – | – | × | – |

### Reproducible gene-expression fingerprints

Total RNA was isolated from Ishikawa cells. The RNA sample was divided in two batches, reverse-transcribed and the cDNA was labeled. The two labeled cDNA batches were hybridized to two twin membranes. A very high $R^2 = 0.92$ was seen (Figure 2a), implying a high level of reproducibility of the cDNA array technique.

### Identification of similarity

All the hybridization experiments were evaluated in pairs (Figure 2). R-squared values (= Pearson correlation coefficient) were calculated. The $R^2$-value represents the variation of data around the least-squares regression line. Therefore the $R^2$-value can be considered as a quantification of similarity between compared data. Table 1 gives a summary of the $R^2$-values, which show

© 2000 Cancer Research Campaign 

interesting trends. Comparing the benign endometrial tissue sample with the two endometrial carcinoma tissue samples resulted in $R^2 = 0,74$ and $R^2 = 0.41$ respectively. A higher degree of similarity in the gene-expression fingerprint was found between the endometrial samples (tissue and cell-line) (average $R^2 = 0.36 \pm 0.13$) than between the endometrial samples (tissue and cell-line) and the breast cancer cell-line (average $R^2 = 0.12 \pm 0.12$), implicating the existence of a tissue-specific gene-expression fingerprint.

### Specific gene expression

Using the xy-scatter plot and 95% prediction interval around the least-squares regression line, differentially expressed genes were identified. Comparing the benign endometrial tissue gene-expression fingerprint with the gene-expression fingerprints taken from endometrial carcinoma tissue and cell-lines, three consistently differentially expressed genes were identified (Table 2). TIMP3 and Decorin are down-regulated in all the carcinoma samples (tissue samples and cell-lines), Cyclin D1 is up-regulated in the carcinoma samples, except for the endometrial carcinoma tissue-sample 54 (Figure 2b and 2c). These three genes may represent important genes involved in the progression of endometrial carcinomas. Another group of genes, which showed expression at similar levels in all endometrial samples, was identified (Table 2). None of these genes was expressed in the human breast cancer cell-line T47D. These genes seem to represent endometrial house-keeping genes. Comparing the breast cancer cell-line T47D with the endometrial carcinoma samples (tissue and cell-line) (Figure 2d) revealed too many differences ($R^2 = 0.12 \pm 0.12$, Table 1) in gene-expression pattern, therefore identification of differential expressed genes was not possible (see Discussion).

### DISCUSSION

The expression level of all the 588 genes on the cDNA expression array plus the 21 positive and negative control dots were used in the analysis. The majority of the gene-expression level signals did not exceed two times the background hybridization signal level (Figure 1). In the xy-scatterplot the least-squares regression line will be highly determined by these signal levels. As long as a high degree of similarity exists between the expression pattern of the array experiments, the slope value a (in the least-squares regression line $y = ax + b$) can be considered to represent the differences in hybridization intensity and the intercept value b to represent the differences in background signal. Therefore, raw data obtained from cDNA expression array hybridization experiments can be analysed without the need of preceding normalization of the data (Figure 2a–c). In the same instance, differentially expressed genes can be identified using a 95% prediction interval around the least-squares regression line. The $R^2$-value of the least-squares regression line quantifies the degree of similarity. However, when little similarity exists between the compared expression patterns, resulting in a low $R^2$-value, the least-squares regression line will be almost random. An example of this situation is seen in the comparison between the gene-expression fingerprint of the T47D cell-line and the endometrial carcinoma tissue sample (Figure 2d). Differentially expressed genes cannot be identified in this case. The lowest $R^2$-value at which the least-squares regression line and 95% prediction interval around the regression line can be used to identify differentially expressed genes has yet to be established.

A higher degree of resemblance exists between the endometrial samples (tissue and cell-line samples), than between the endometrial samples and the breast cancer cell-line, implicating a basic endometrial gene-expression fingerprint. As tumour behaviour is thought to be driven by genetic alterations, it is plausible that clinical behaviour of carcinomas can be reflected in their specific gene-expression fingerprints (Blok et al, 1995; 1999; Chang et al, 1997). Taking this one step further, a database could be constructed containing gene-expression fingerprints of various endometrial carcinoma tissue samples of different clinical stage, different histological grade and different progesterone and oestrogen receptor status linked to information on clinical follow-up. Comparing the gene-expression fingerprint of an uncharacterized tumour sample of interest with the database could reveal the potential clinical behaviour of the uncharacterized tumour. A table of $R^2$-values, as shown in Figure 2, could be used to establish the degree of similarity between the tumour samples. Using the cDNA expression array to reveal degrees of similarity between uncharacterized tumour samples of interest and tumour samples of known clinical behaviour, could have great potential value in the diagnosis and prognosis of endometrial cancer. However, to confirm the potential value of the cDNA arrays as a diagnostic and prognostic tool, a gene-expression fingerprint database of a large group of fully characterized endometrial carcinoma tissue samples should be constructed and analysed. The current investigation may provide a practical and useful method to analyse these data.

Comparing gene-expression fingerprints of different tumour samples will also identify differentially expressed genes. Using the xy-scatterplot analysis and the 95% prediction interval around the least-squares regression line, three genes (Decorin, TIMP3 and Cyclin D1) were identified to be differentially expressed between benign endometrial cells and endometrial carcinoma cells. Decorin (small leucin-rich protoglycan) inhibits TGFβ and induces p21, resulting in inhibition of proliferation (Stander et al, 1999; Iozzo et al, 1999). TIMP3 (tissue inhibitor of metaloproteinase number 3) inhibits the matrix metaloproteinases MMPs. Excess of MMPs stimulates tumour invasion and metastasis (Kugler, 1999; Sato et al, 1992). Cyclin D1 initiates the G1-S phase progression (Chen et al, 1998). Oestrogens are found to induce nuclear localization of Cyclin D1 in endometrial cells, resulting in increased cell proliferation (Tong and Pollard, 1999). In breast cancers Cyclin D1 can act as an oncogene (Russell et al, 1999) and is found to act as a CDK-independent activator of the oestrogen receptor in breast epithelial cells (Zwijsen et al, 1997). The loss of Decorin and TIMP3 expression and the gain of Cyclin D1 expression in the endometrial carcinoma cell-lines and tissue samples could indicate an important role of Decorin, TIMP3 and Cyclin D1 in the development and progression of endometrial cancer. However, the number of samples analysed is too small to draw conclusions.

### ACKNOWLEDGEMENTS

We thank Dr Alberda and the Department of Obstetrics and Gynaecology of Sint Franscicus Hospital (Rotterdam, The Netherlands) for the gift of human endometrial carcinoma tissue samples. We are grateful to Dr Masato Nishida (Japan) for the gift of the Ishikawa cells, to Dr PG Satyaswaroop (USA) for the gift of the ECC-1 cells and Dr B van der Burg (Utrecht, Netherlands) for the gift of the T47D cells. This work was supported by a grant from The Netherlands Organisation for Scientific Research (NWO 903–46–169) and by the Erasmus Trust Funds.

*© 2000 Cancer Research Campaign*

### REFERENCES

Blok LJ, Grossmann ME, Perry JE and Tindall DJ (1995) Characterization of an early growth response gene, which encodes a zinc finger transcription factor potentially involved in cell cycle regulation. *Mol Endocrinol* **9**: 1610–1620

Blok LJ, Chang GT, Steenbeek-Slotboom M, van Weerden WM, Swarts HG, De Pont JJ, van Steenbrugge GJ and Brinkmann AO (1999) Regulation of expression of $Na^+$, $K^+$-ATPase in androgen-dependent and androgen-independent prostate cancer. *Br J Cancer* **81**: 28–36

Chang GTG, Blok LJ, Steenbeek M, Veldscholte J, van Weerden WM, van Steenbrugge GJ and Brinkmann AO (1997) Differentially expressed genes in androgen-dependent and androgen-independent prostate carcinomas. *Cancer Res* **57**: 4075–4081

Chen Y, Martinez LA, LaCava M, Coghlan L and Conti CJ (1998) Increased cell growth and tumorigenicity in human prostate LNCaP cells by overexpression to cyclin D1 *Oncogene* **16**: 1913–1920

Fearon ER and Vogelstein BA (1990) A genetic model for colorectal tumorigenesis. *Cell* **61**: 759–767

Hilsenbeck SG, Friedrichs WE, Schiff R, O'Connell P, Hansen RK, Osborne CK and Fuqua SAW. (1999). Statistical analysis of array expression data as applied to the problem of tamoxifen resistance. *J Natl Cancer Inst* **91**: 453–459

Hoch RV, Thompson DA, Baker RJ and Wiegel RJ (1999) GATA-3 is expressed in association with estrogen receptor in breast cancer. *Int J Cancer* **84**: 122–128

Iozzo RV, Chakrani F, Perrotti D, McQuillan DJ, Skorski T, Calabretta B and Eichstetter I (1999) Cooperative action of germ-line mutations in decorin and p53 accelerates lymphoma tumorigenesis. *Proc Natl Acad USA* **96**: 3092–3097

Kaiser A, Brembeck FH, Marschall Z, Wiedenmann B and Rosewicz S (1999) Fra-1: a novel target for retinoid action. *FEBS Letters* **448**: 45–48

Knudson AG (1993) All in the (cancer) family. *Nat Genet* **5**: 103–104

Kugler A (1999) Matrix metalloproteinases and their inhibitors. *Anticancer Res* **19**: 1589–1592

Lentz SS (1994) Advanced and recurrent endometrial carcinoma: hormonal therapy. *Semin Oncol* **21**: 100–106

Mikuta JJ (1993) International Federation of Gynecology and Obstetrics staging of endometrial cancer 1988. *Cancer* **71**: 1460–1463

Rhee CH, Hess K, Jabbur J, Ruiz M, Yang Y, Chen S, Chenchik A, Fuller GN and Zhang W (1999) cDNA expression array reveals heterogeneous gene expression profiles in three glioblastoma cell lines. *Oncogene* **18**: 2711–2717

Rose PG (1996) Endometrial carcinoma. *New Engl J Med* **335**: 640–649

Russell A, Thompson MA, Hendly J, Trute L, Armes J and Germain D (1999) Cyclin D1 and D3 associate with the SCF complex and are coordinately elevated in breast cancer. *Oncogene* **18**: 1983–1991

Sato H, Kida Y, Mai M, Endo Y, Sasaki T, Tanaka J and Seiki M (1992) Expression of genes encoding type IV collagen-degrading metalloproteinases and tissue inhibitors of metalloproteinases in various human tumor cells. *Oncogene* **7**: 77–83

Schottenfeld D (1995) Epidemiology of endometrial neoplasia. *J Cell Biochem* **23**: 151–159

Shim C, Zhang W, Rhee CH and Lee JH (1998) Profiling of differentially expressed genes in human primary cervical cancer by complementary DNA expression array. *Clin Cancer Res* **4**: 3045–3050

Stander M, Naumann U, Wick W and Weller M (1999) Transforming growth factor-beta and p-21: multiple molecular targets of decorin-mediated suppression of neoplastic growth. *Cell Tissue Res* **296**: 221–227

Tong W and Pollard JW (1999) Progesterone inhibits estrogen-induced cyclin D1 and cdk4 nuclear translocation, cyclin E-and cyclin A-cdk2 kinase activation, and cell proliferation in uterine epithelial cells in mice. *Mol Cell Biol* **19**: 2251–2264

Zwijsen RM, Wientjes E, Klompmaker R, van der Sman J, Bernards R and Michalides RJ (1997). CDK-independent activation of estrogen receptor by cyclin D1. *Cell* **88**: 405–415

*© 2000 Cancer Research Campaign*

## References

[PDF_00466] Blok L. J., Chang G. T., Steenbeek-Slotboom M., van Weerden W. M., Swarts H. G., De Pont J. J., van Steenbrugge G. J., Brinkmann A. O. (1999). Regulation of expression of Na+,K+-ATPase in androgen-dependent and androgen-independent prostate cancer.. Br J Cancer.

[PDF_00463] Blok L. J., Grossmann M. E., Perry J. E., Tindall D. J. (1995). Characterization of an early growth response gene, which encodes a zinc finger transcription factor, potentially involved in cell cycle regulation.. Mol Endocrinol.

[PDF_00472] Chang G. T., Blok L. J., Steenbeek M., Veldscholte J., van Weerden W. M., van Steenbrugge G. J., Brinkmann A. O. (1997). Differentially expressed genes in androgen-dependent and -independent prostate carcinomas.. Cancer Res.

[PDF_00481] Hilsenbeck S. G., Friedrichs W. E., Schiff R., O'Connell P., Hansen R. K., Osborne C. K., Fuqua S. A. (1999). Statistical analysis of array expression data as applied to the problem of tamoxifen resistance.. J Natl Cancer Inst.

[PDF_00484] Hoch R. V., Thompson D. A., Baker R. J., Weigel R. J. (1999). GATA-3 is expressed in association with estrogen receptor in breast cancer.. Int J Cancer.

[PDF_00486] Iozzo R. V., Chakrani F., Perrotti D., McQuillan D. J., Skorski T., Calabretta B., Eichstetter I. (1999). Cooperative action of germ-line mutations in decorin and p53 accelerates lymphoma tumorigenesis.. Proc Natl Acad Sci U S A.

[PDF_00489] Kaiser A., Brembeck F. H., v Marschall Z., Riecken E. O., Wiedenmann B., Rosewicz S. (1999). Fra-1: a novel target for retinoid action.. FEBS Lett.

[PDF_00491] Knudson A. G. (1993). All in the (cancer) family.. Nat Genet.

[PDF_00492] Kugler A. (1999). Matrix metalloproteinases and their inhibitors.. Anticancer Res.

[PDF_00494] Lentz S. S. (1994). Advanced and recurrent endometrial carcinoma: hormonal therapy.. Semin Oncol.

[PDF_00496] Mikuta J. J. (1993). International Federation of Gynecology and Obstetrics staging of endometrial cancer 1988.. Cancer.

[PDF_00498] Rhee C. H., Hess K., Jabbur J., Ruiz M., Yang Y., Chen S., Chenchik A., Fuller G. N., Zhang W. (1999). cDNA expression array reveals heterogeneous gene expression profiles in three glioblastoma cell lines.. Oncogene.

[PDF_00502] Rose P. G. (1996). Endometrial carcinoma.. N Engl J Med.

[PDF_00503] Russell A., Thompson M. A., Hendley J., Trute L., Armes J., Germain D. (1999). Cyclin D1 and D3 associate with the SCF complex and are coordinately elevated in breast cancer.. Oncogene.

[PDF_00506] Sato H., Kida Y., Mai M., Endo Y., Sasaki T., Tanaka J., Seiki M. (1992). Expression of genes encoding type IV collagen-degrading metalloproteinases and tissue inhibitors of metalloproteinases in various human tumor cells.. Oncogene.

[PDF_00510] Schottenfeld D. (1995). Epidemiology of endometrial neoplasia.. J Cell Biochem Suppl.

[PDF_00512] Shim C., Zhang W., Rhee C. H., Lee J. H. (1998). Profiling of differentially expressed genes in human primary cervical cancer by complementary DNA expression array.. Clin Cancer Res.

[PDF_00515] Ständer M., Naumann U., Wick W., Weller M. (1999). Transforming growth factor-beta and p-21: multiple molecular targets of decorin-mediated suppression of neoplastic growth.. Cell Tissue Res.

[PDF_00518] Tong W., Pollard J. W. (1999). Progesterone inhibits estrogen-induced cyclin D1 and cdk4 nuclear translocation, cyclin E- and cyclin A-cdk2 kinase activation, and cell proliferation in uterine epithelial cells in mice.. Mol Cell Biol.

[PDF_00522] Zwijsen R. M., Wientjens E., Klompmaker R., van der Sman J., Bernards R., Michalides R. J. (1997). CDK-independent activation of estrogen receptor by cyclin D1.. Cell.

